# Effects of P-15 Peptide Coated Hydroxyapatite on Tibial Defect Repair In Vivo in Normal and Osteoporotic Rats

**DOI:** 10.1155/2015/253858

**Published:** 2015-10-05

**Authors:** Rasmus Hestehave Pedersen, Marina Rasmussen, Søren Overgaard, Ming Ding

**Affiliations:** Orthopaedic Research Laboratory, Department of Orthopaedic Surgery and Traumatology, Odense University Hospital, Institute of Clinical Research, University of Southern Denmark, 5000 Odense C, Denmark

## Abstract

This study assessed the efficacy of anorganic bone mineral coated with P-15 peptide (ABM/P-15) on tibia defect repair longitudinally in both normal and osteoporotic rats in vivo. A paired design was used. 24 Norwegian brown rats were divided into normal and osteoporotic groups. 48 cylindrical defects were created in proximal tibias bilaterally. Defects were filled with ABM/P-15 or left empty. Osteoporotic status was assessed by microarchitectural analysis. Microarchitectural properties of proximal tibial defects were evaluated at 4 time points. 21 days after surgery, tibias were harvested for histology and histomorphometry. Significantly increased bone volume fraction, surface density, and connectivity were seen in all groups at days 14 and 21 compared with day 0. Moreover, the structure type of ABM/P-15 group was changed toward typical plate-like structure. Microarchitectural properties of ABM/P-15 treated newly formed bones at 21 days were similar in normal and osteoporotic rats. Histologically, significant bone formation was seen in all groups. Interestingly, significantly increased bone formation was seen in osteoporotic rats treated with ABM/P-15 indicating optimized healing potential. Empty defects showed lower healing potential in osteoporotic bone. In conclusion, ABM/P-15 accelerated bone regeneration in osteoporotic rats but did not enhance bone regeneration in normal rats.

## 1. Introduction

One of the major clinical challenges, in orthopedic and reconstructive surgeries, is filling large osseous defects [1, 2]. Bone defects can be due to trauma, surgical procedures, tumor resection, or age-related skeletal diseases such as osteoporosis [3]. Osteoporosis (OP) is a skeletal disorder affecting bone tissue and is characterized by loss of bone mass to a critical level, for bone fractures. Osteoporosis is a public health care problem because of the increase in elderly population, especially elderly women, who suffer additional bone loss due to menopause [3, 4]. In this study an osteoporotic rat model was used to mimic reduced bone quality as found in elderly patients, who are characterized by bone loss related increased fracture risk and decreased fracture healing potential [5].

Large bone defects require bone grafting in order to get healing [6, 7]. Autograft has been the preferred graft material and considered as gold standard. However, there are several difficulties connected to the use of autograft material, for example, getting adequate amount of material and morbidity at donor site [7–10]. Furthermore the current allograft and xenograft products might cause graft versus host reaction, because the histocompatibility antigens from the graft are different from those of the host [9].

Because of the problems associated with autograft, allograft, or xenograft, bioactive graft materials are getting increasing attention. These bone substitutes have attracted great interest because of their potential to be a substitute for autogenous bone, with no supply limits and low risk of adverse effects [7].

The combination of anorganic bone mineral (ABM/P-15) and synthetic peptide P-15 acts as a bioactive attachment factor that aims to replicate type I collagen binding mechanism with osteogenic precursor cells in bone tissue [11, 12]. The ABM/P-15 has been tested in several animal studies, presenting ABM/P-15 with properties similar to those of autogenous bone [10, 13]. The effectiveness of ABM/P-15 in clinical dental use has been shown in a few studies [14, 15]. The efficacy of ABM/P-15 in orthopedics has been addressed in a number of animal studies [16, 17] but only one clinical study has been done in human [18]. However, there is no published literature on the effects of ABM/P-15 on bone defect repair in vivo in particular osteoporotic model longitudinally.

It was hypothesized that ABM/P-15 would accelerate bone regeneration in both normal and osteoporotic bones. Thus the purpose of this study was to assess the efficacy of ABM/P-15 on bone healing in normal rat and osteoporotic rat defects, evaluated as histomorphometry on bone formation and changes in microarchitecture.

## 2. Materials and Methods

### 2.1. ABM/P-15

Anorganic bone mineral is a microporous bovine-derived hydroxyapatite. The P-15 peptide is synthetic replica of the (766)GTPGPQGIAGQRGVV(780) sequence in the *α*(I)chain of type I collagen. The P-15 sequence has been shown to induce bone healing, similar to type I collagen [13]. When bound to anorganic bone mineral, P-15 peptide serves as a surrogate for collagen [19]. P-15 interacts with osteogenic precursor cells, which contains *α*2*β*1-integrins. The binding of *α*2*β*1-integrins to P-15 initiates natural intra- and extracellular signaling pathways, inducing the production of growth factors, bone morphogenic proteins [20], and cytokines. Ultimately, the injection of ABM/P-15 into a bony defect site might initiate new bone formation and optimize the natural bone healing process [13]. ABM/P-15 (i-Factor Putty, Cerapedics Inc., Westminster, USA) substitute was donated by Ortotech (Kolding, Denmark), delivered in form of putty in 5.0 cc syringes.

### 2.2. Animals

Twenty-four female brown Norwegian inbred rats (BN/SsNOlaHsd), 4 months of age, with a mean body weight of 194.5 ± 10.1 grams, were purchased from Harlan Laboratories GmbH (Venray, Netherlands) and used in this study. These rats were housed in the Biomedical Laboratory Facility, University of Southern Denmark and were acclimated for two weeks prior to the experimental initiation. The environment was temperature-controlled (21°C ± 2°C/40–60% humidity). The normal rats received standard food, and the ovariectomized rats were given a phytoestrogen-free calcium deficient diet containing <0.15% calcium and <0.1% phosphorus (Brogaarden, Lynge, Denmark) starting two months before tibial defects were created. Body weight was recorded over time, and physical activities were observed daily. Housing conditions and applied experimental protocol were in accordance with Danish Animal Research guidelines and approved by the Danish Animal Experiments and Inspectorates, with number 2011/561-1959.

### 2.3. Study Design

**Figure 1:**
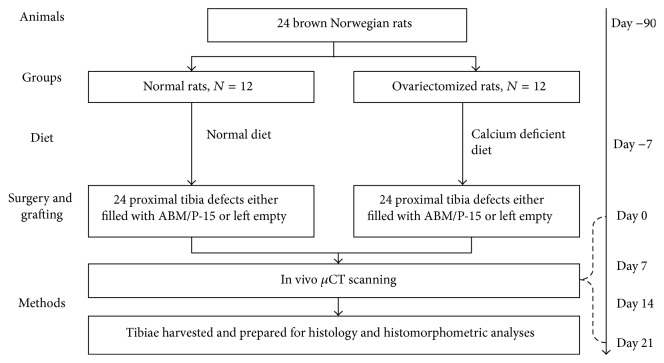
Study design. Schematic flowchart drawing illustrates the study design and the preparation of the bone samples. After in vivo *μ*CT scanning at four time points, 42 tibias were harvested from 21 rats. The proximal ends of each tibia containing the defect site were decalcified and embedded in paraffin for histomorphometric analyses.

The schematic drawing in Figure 1 illustrates the study designs. The animals were divided into two experimental groups. Group 1 had twelve normal rats, and group 2 had twelve osteoporotic rats. In both groups, the effects between ABM/P-15 and empty control on defect healing were compared. Side location of ABM/P-15 and empty defect in left or right proximal tibia was randomized. After in vivo scanning, all tibias were harvested and prepared for histologic and histomorphometric analyses (Figure 1). Specific procedures are described below.

### 2.4. Surgical Procedures

Surgical procedures were performed at the Biomedical Laboratory, University of Southern Denmark.

#### 2.4.1. Ovariectomy (OVX)

Animals were anesthetized with 0.3 mL/100 g of bodyweight of Hypnorm (VetaPharma Ltd., Leeds, UK) and Midazolam (B. Braun, Melsungen, Germany) mixture subcutaneously. Initial analgesics were given, 0.2 mL/100 g of bodyweight of Temgesic (RB Pharmaceuticals Limited, Berkshire, UK). Once anesthetized, the animals were shaved on the lumbar part of the back. A midline dorsal skin incision was made. Blunt dissection of the connective tissue between skin and muscle layer gave access to the abdominal wall. The muscular layer was perforated 2 centimetres lateral of the dorsal midline to gain access to the abdominal cavity. The ovary, located surrounded by a large fat pad, was pulled out the incision and fixated. Two ligatures of absorbable 5.0 ethilon sutures were placed between the uterine horn and the ovary. The ovary was safely cut off and the uterine horn was placed back into the abdominal cavity. The muscle layer was closed with absorbable 5.0 sutures. Bilateral ovary resection was performed from the same initial median incision. After resection, the wound was carefully closed in layers with 4.0 ethilon sutures. After surgery, analgesic, Temgesic 0.2 mL/100 g of bodyweight, was administered subcutaneously three times a day for three days.

#### 2.4.2. Tibial Surgery for Creating Defect (Called “Surgery” Below)

These animals were anesthetized by the same method as described above. When anaesthesia was achieved, both hind limbs were shaved. An incision, approximately 8 mm in length was made on medial proximal tibia. Blunt dissection of connective tissue gave access to the medial surface of proximal tibia. A defect was created with a 2 mm diameter k-wire and a 2.8 diameter drill in a frontal plane from medial side towards the lateral cortical shell. The cylindrical defects had a diameter of 2.8 mm and a depth of 3 mm, thus a total volume of 18.47. Care was taken not to drill through the lateral cortical shell. The defect was cleansed for bone remnants and blood. The defect was either filled with ABM/P-15 or left empty. After filling the defect, the wound was closed in layers with 4.0 ethilon sutures. In total, forty-eight proximal tibial cylindrical defects were created in twenty-four rats. Analgesics, Temgesic 0.2 mL/100 g of bodyweight, were administered three times a day for 4 days postoperatively. The animals were allowed to move without any restrictions.

### 2.5. Micro-CT Scanning and Microarchitectural Analysis

All rats were scanned in vivo with a high-resolution micro computed tomographic system (vivaCT40, Scanco Medical AG, Brüttisellen, Switzerland) using 55 kVp and 72 *μ*A. During the induction of osteoporosis period, the osteoporotic rats were in vivo micro-CT scanned at 3 time points: (1) right after ovariectomy at the same day (defined as week day −90, that is, 3 months before tibia surgery); (2) one week before the tibia surgery (day −7); (3) day of surgery (day 0, preoperatively). During the defect healing period, both groups were scanned with in vivo micro-CT at four time points, immediately following surgery at the same day serving as baseline (day 0, postoperatively), day 7, day 14, and day 21 postoperatively, respectively.

All scans were based on the same control file, and the same region of interest was scanned. During in vivo scanning, the rat was anesthetized using Isoflurane (IsoFlo vet, Abbott Laboratories Ltd., Berkshire, England). The rat was initially placed in a chamber filled with isoflurane to reach acceptable anaesthetic condition, and the anaesthesia was maintained with a mask during scanning process. Each scan took approximately 30 min and created 381 micro-CT images slices. All scanned micro-CT images resulted in 3D reconstruction cubic voxel sizes of 10.5 × 10.5 × 10.5 *μ*m^3^ (2048*∗*2048*∗*2048 pixels) with 32-bit gray levels. The images were segmented using the segmentation techniques described in detail previously [21, 22] to obtain accurate 3D imaging datasets.

The changes of microarchitectural properties in proximal tibial cortical bone during development of osteoporosis were evaluated. These parameters were cortical porosity (%), bone surface density (mm^−1^), bone surface to volume ratio (mm^−1^), pore size (*μ*m), and cortical thickness (*μ*m).

The changes of microarchitectural parameters in proximal tibial cancellous bone during development of osteoporosis were also quantified. These parameters were bone volume fraction (BV/TV) [23], bone surface density (BS/TV), bone surface-to-volume ratio (BS/BV), structure model index (SMI), connectivity density (mm^−3^), trabecular separation (TbSp^*∗*^), degree of anisotropy (—), and trabecular thickness (TbTh^*∗*^).

The progress of defect healing bone mass was evaluated similar to above description for cancellous bone.

### 2.6. Histomorphometry

Three weeks after surgery, rats were euthanized using CO_2_ and the tibiae were harvested and prepared for further histology and histomorphometry.

Stereological histomorphometry was used to quantify volume fractions of tissue inside the defect area using an Olympus BX 51 Microscope (Ballerup, Denmark). Bone samples were fixed in 4% formaldehyde. Specimens were dehydrated in graded ethanol 77–96% and then paraffin embedded for classical histomorphometry of bone formation parameters [24]. Each bone sample was sawed into 3 sections of 3 sectioning levels (4 *μ*m thickness with 500 *μ*m separation between each sectioning level, thus 9 sections per specimen). All sections were stained with haematoxylin and eosin.

Stereological software (newCAST, Visiopharm, Denmark) was used to quantify volume fractions in predefined regions of interest (ROI), being the defect area. Volume fractions of bone, fibrous tissue, ABM/P-15 remnants, and marrow cavity were estimated by using point-counting technique [25]. Three sections were chosen in order to reduce the variance of the estimates [26]. The quantified area fraction of bone was defined as newly formed bone. Micro-CT could not distinguish between bone and ABM/P-15 remnants in the defect area. This was taken into account when comparing with micro-CT data.

### 2.7. Statistical Analysis

All results are expressed as mean for group ± SD. One-way ANOVA and Friedman test were used to calculate possible differences between groups. Post hoc multiple comparisons were adjusted using Bonferroni test or Dunnett's test as appropriate for normal and nonnormal distributed data. Histomorphometric parametric data was analyzed with Student's paired *t*-test or nonparametric data with Wilcoxon Rank as appropriate. *P* value less than 5% was considered significant.

## 3. Results

### 3.1. Animals

The mean weight gain of the animals in the osteoporotic group was 48.4 grams (SD = 34.2 grams) in contrast to the normal group, which showed a nonsignificant mean weight gain of 28.0 grams (SD = 34.5 grams) during the study period.

Three animals were lost from the normal group during the *μ*CT scanning period due to anaesthesia, leaving nine rats for further analysis. No animals were lost in the osteoporotic group. Three sections in normal group and two sections in osteoporotic group were damaged during the preparation procedure, were unusable for histological analysis, and were excluded. Nine normal group rats and twelve osteoporotic group rats were included in the analysis.

### 3.2. Microarchitectural Analysis of Osteoporotic Bone during the Induction Period

**Figure 2:**
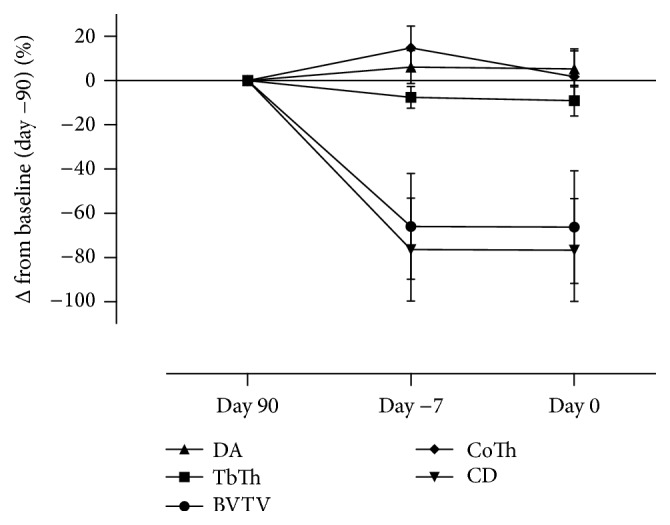
Microarchitectural changes during the induction of osteoporosis. Graphic presentation of the development of osteoporosis in ovariectomized rats. 12 rats were ovariectomized three months prior to the surgery for creating defect. Bone loss was expressed as mean for each parameter. Significantly deteriorated microarchitecture could be observed after 11 weeks, with reduction in bone volume fraction (BV/TV) 65.5% and connectivity density (CD) 77.5%, and more importantly these changes remained constant. Cortical thickness (CoTh), trabecular thickness (TrTh), and degree of anisotropy (DA).

The animals in the OVX group showed marked decreases in bone volume fraction (65.8%, *P* < 0.0001) and connectivity density (77.8%, *P* < 0.0001), indicating a marked loss of cancellous bone during the induction period prior to surgery, that is, three months after OVX (Figure 2). Decrease was also seen in bone surface density (57.1%, *P* < 0.0001) and trabecular thickness (9.1%, *P* = 0.0006) three months after OVX. In contrast significant increases were seen in bone surface-to-volume ratio (40.4%, *P* = 0.0001), structure model index (252.2%, *P* < 0.0001), and trabecular separation (118.9%, *P* = 0.0017) three months after OVX. Degree of anisotropy showed only significant increase between day −90 and day −7 before surgery (5.9%, *P* = 0.0265) (Table 1).

For cortical bone, cortical porosity was not changed (18.2% reduction, *P* = 0.1794) three months after OVX (Figure 2 and Table 2). Seven days before surgery, significant decreases were seen in bone surface density (24.3%, *P* = 0.0001) and bone surface-to-volume ratio (22.2%, *P* = 0.0001). Significant increase was also seen for pore size (66.7%, *P* = 0.0018) after three months. Cortical thickness showed a small increase after three months (1.8%, *P* = 0.0003) (Table 2).

### 3.3. Microarchitectural Analysis of Calcified Material in Cylindrical Defect Region

#### 3.3.1. Changes in Microarchitecture in Normal Rats

Seven days after surgery, degree of anisotropy decreased for the empty group (*P* = 0.002), but the ABM/P-15 group showed increase from day seven to day fourteen (*P* = 0.037).

**Figure 3:**
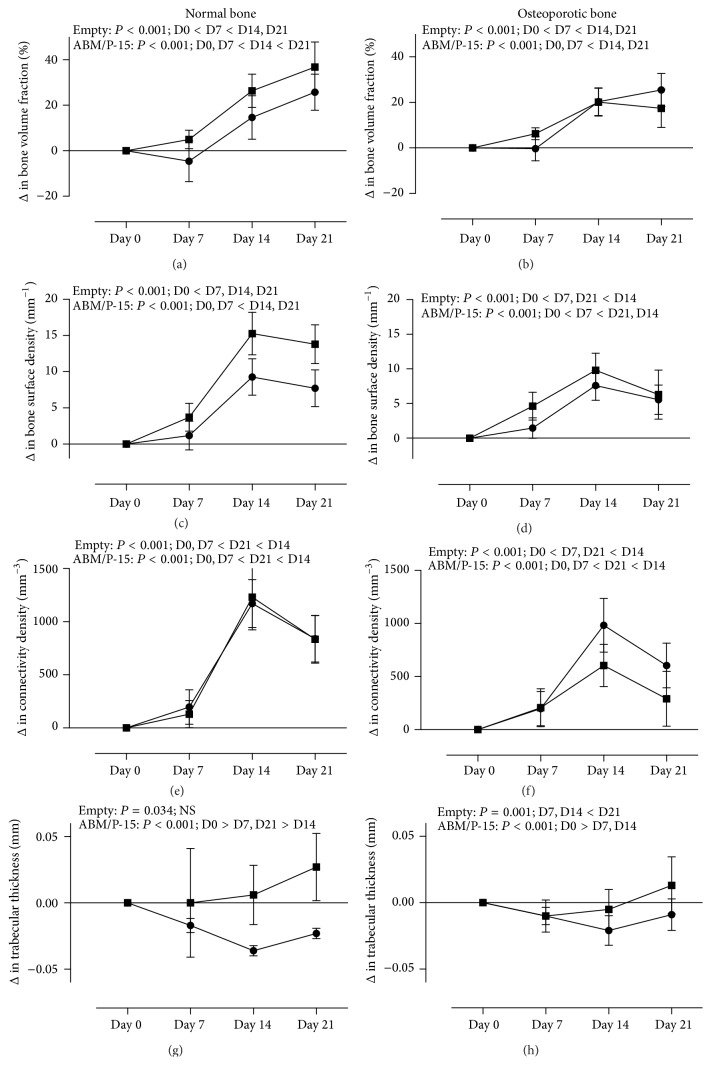
Microarchitectural changes after surgery for creating defect in the normal and the osteoporotic rats. Microarchitectural parameters of the tibial defect areas in normal and osteoporotic rats are presented. Square points represent values from empty groups. Round points represent ABM/P-15 groups. Values are expressed as mean ± SD for groups and are depicted in normal group (a, c, e, g) and in osteoporotic group (b, d, f, h).

Fourteen days after surgery, bone surface density (*P* < 0.001) and connectivity density (*P* < 0.001) increased for both the empty group and the ABM/P-15 group. Only ABM/P-15 group decreased in trabecular thickness (*P* < 0.001) (Figure 3).

Twenty-one days after surgery, bone volume fraction increased for both the empty group (*P* < 0.001) and the ABM/P-15 group (*P* < 0.001) (Figure 3). Structure model index decreased for the empty group (*P* < 0.001) and for the ABM/P-15 group (*P* < 0.001). Trabecular separation decreased for the empty group (*P* < 0.001) and for the ABM/P-15 group (*P* < 0.001).

Bone surface-to-volume ratio decreased for the empty group (*P* < 0.001) after twenty-one days but increased after fourteen days from 28.3 mm^−1^ to 39.6 mm^−1^ for the ABM/P-15 group (*P* < 0.001) (Table 3).

#### 3.3.2. Changes in Microarchitecture in Osteoporotic Rats

Seven days after surgery, trabecular thickness decreased for the empty group (*P* < 0.001) (Figure 3). Degree of anisotropy decreased only for the empty group (*P* = 0.001).

Fourteen days after surgery bone surface density (*P* < 0.001) and connectivity density (*P* < 0.001) increased for both the empty group and the ABM/P-15 group. Bone volume fraction increased for the empty group (*P* < 0.001) (Figure 3).

Structure model index decreased after fourteen days, for the empty group (*P* < 0.001). Trabecular separation decreased both for the empty group (*P* < 0.001) and for the ABM/P-15 group (*P* < 0.001). A decrease in trabecular thickness for the ABM/P-15 group (*P* < 0.001) was seen after fourteen days (Figure 3).

Twenty-one days after surgery, bone surface-to-volume ratio was decreased only for the empty group (*P* < 0.001). An increase in bone volume fraction for the ABM/P-15 group was seen after twenty-one days (*P* < 0.001). Structure model index decreased after twenty-one days, for the ABM/P-15 group (*P* < 0.001) (Table 4).

### 3.4. Histology

**Figure 4:**
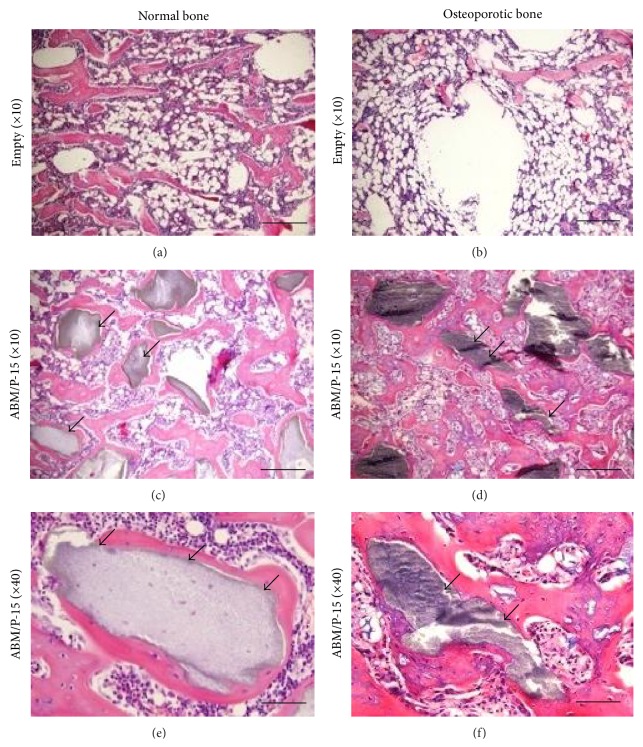
Histological images of defect repairs for both normal and osteoporotic rats. Histology images of defects in normal bone (a, c, e) and osteoporotic bone (b, d, f) are shown. Black arrows indicate residual ABM/P-15 particles inside the defect areas. Size lines in bottom right corner represent 1 mm (a–d) and 250 *μ*m (e and f).

Defects in the ABM/P-15 group showed obvious remodelling and new bone formation in direct apposition to ABM/P-15 in both normal and osteoporotic rats. Most of the remnant ABM/P-15 particles in the defects were completely surrounded by newly formed bone. The contact surfaces between newly formed bone and ABM/P-15 particles were in general solid and continuous (Figure 4). The defects that were left empty showed considerable more bone growth in normal bone than in osteoporotic bone.

### 3.5. Histomorphometry

**Figure 5:**
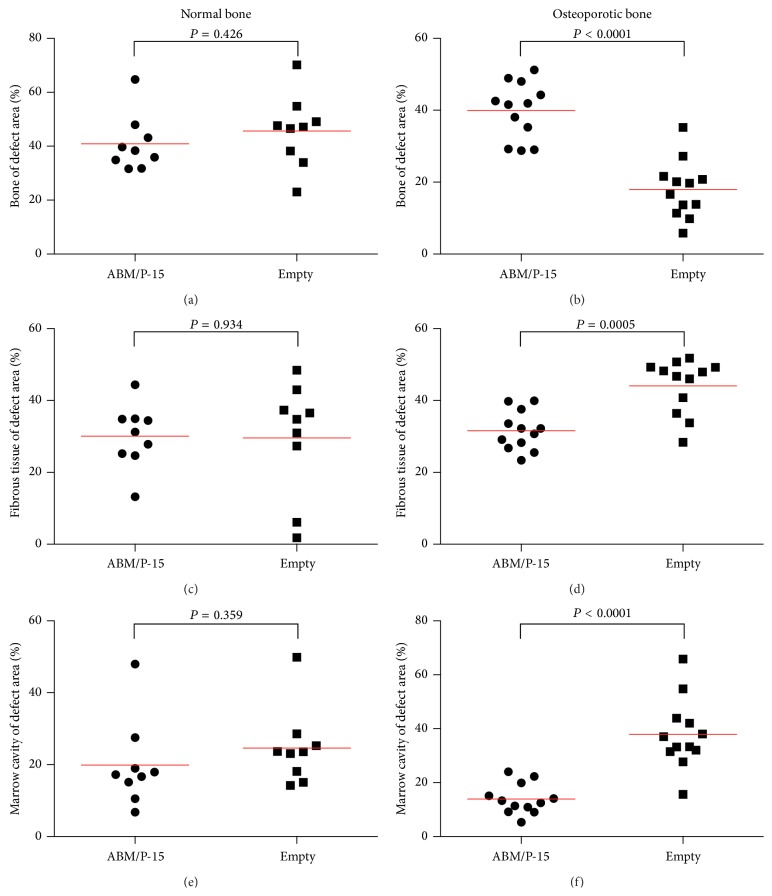
Results from histomorphometrical analysis of defect repairs for both normal and osteoporotic rats. Results of histomorphometric analysis for normal and osteoporotic rats 21 days after surgery are presented. Each dot represents mean value of three measurements 500 *μ*m apart in one defect. Horizontal lines express mean for group.

There was no difference in new bone, fibrous tissue, residual unabsorbed ABM/P-15 or marrow cavity, between the empty group and the ABM/P-15 group, in normal rats (Figure 5).

In osteoporotic rats, the empty group had 17.7% of new bone in the defect area, which was significantly less than 39.9% new bone in the ABM/P-15 group (paired *t*-test *P* < 0.0001). The empty group showed 43.8% fibrous tissue and 38.4% marrow cavity in the defect area, which both were significantly more than 31.6% fibrous tissue (paired *t*-test *P* = 0.0005) and 13.9% marrow cavity (paired *t*-test *P* < 0.0001) in the ABM/P-15 group. 14.6% residual unabsorbed ABM/P-15 was seen in the ABM/P-15 group.

## 4. Discussion

In this study, the effects of ABM/P-15 on bone defect healing were investigated in tibia defect models in both normal and osteoporotic rats [27]. The changes in microarchitectural parameters were significant for all groups during the observation period of twenty-one days. Histomorphometry revealed significantly improved defect repair in the osteoporotic ABM/P-15 group compared with the empty group. These results indicate that ABM/P-15 significantly promoted new bone formation in osteoporotic bone but did not accelerate new bone formation in normal bone. These results did support our hypothesis that bone repair in osteoporotic bone was significantly accelerated by ABM/P-15, but not that ABM/P-15 would enhance bone repair in normal bone. Furthermore, histology demonstrated extensive new bone formation adjacent to ABM/P-15 with direct contact between bone and ABM/P-15. These findings suggest that ABM/P-15 grafting material provides osteoconductive and osteoinductive properties, both being important characteristics of an ideal bone graft substitute [28–30].

A few studies have tested the effects of ABM/P-15 on different types of bone defects in small and large animal models, but no longitudinal studies have been done.

We have recently demonstrated the efficacies of ABM/P-15 on bone formation and implant fixation in sheep, and these effects were at least as good as allograft [31]. Scarano et al. [32] showed that ABM/P-15 enhanced new bone regeneration in 8 mm tibial cortical defects in rabbits. The newly formed bone surrounding the residual P-15 particles after 4 weeks was more mature compared with bone formed in untreated empty defects. Wojtowicz et al. [33] found accelerated and increased bone formation in 8 mm segmental femur defect in rats treated with GFOGER, a triple helical peptide that similar to ABM/P-15 binds to the *α*2*β*1-integrin receptor. A clinical pilot study by Gomar et al. [18] showed full consolidation healing in delayed and nonunion extremities fractures in twenty of twenty-two human cases. Full consolidation was determined radiographically as callus formation in the full thickness of the bone.

Some studies have reported that ABM/P-15 did not promote new bone formation. Sarahrudi et al. [34] showed reduced production of new bone in defects treated with ABM/P-15 compared with empty control defects after 8 to 12 weeks, in a 5 mm segmental rabbit femur defect. Mardas et al. [35] demonstrated that ABM/P-15 failed to promote bone healing in critical sized rat calvarial defects, compared with untreated controls. There was no significant difference between treatment group and control group 60 days or 120 days postoperatively.

In the present study the effect of ABM/P-15 on osteoporotic bone defects was evaluated. We observed a decrease in cancellous bone volume fraction of 66.2%, a decrease in trabecular thickness of 9.1%, and a decrease in connectivity density of 77.8% after three months. Cheng et al. [36] showed a 19.5% decrease in bone mineral density of the lumbar spine in a rat after three months. Heiss et al. [37] showed a 15.7% decrease in bone mineral density also after three months. Campbell et al. [38] showed a 37% decrease in cancellous bone volume fraction, in rat proximal tibiae after eight weeks. Furthermore, they showed significant decrease in trabecular thickness and connectivity density. Boyd et al. [39] have observed similar development after three months. In this study cortical thickness showed a 1.8% increase after three months. Similar changes in cortical bone have been reported in earlier studies [38]. The changes in cancellous bone in ovariectomized rats were similar to reported ongoing changes in human normal aging bone and osteoporotic bone [40]. Thus this model was highly clinically relevant.

Histological and histomorphometric analyses did not show improved bone defect healing in normal rats, when comparing the ABM/P-15 group with the empty group after twenty-one days. This supports the finding of an earlier study [34]. One explanation would be that the defect area was too small (2.8 mm in diameters) to expect impaired bone regeneration in normal bone. Interestingly, bone volume fraction of the ABM/P-15 group in osteoporotic rats reached the same level as the ABM/P-15 group in normal rats indicating that ABM/P-15 may optimize the healing potential in osteoporotic bone. Histomorphometry results showed similar fractions of bone in the defect areas of the ABM/P-15 treated groups in both normal and osteoporotic rats. In osteoporotic rats, the bone repair potential was impaired. It was thus of great interest to see a significant improvement of bone formation in the ABM/P-15 group compared with the empty group (*P* < 0.001) in osteoporotic bone. Slight decreases in bone volume fractions were observed in both ABM/P-15 groups after seven days. An initial inflammatory process, as described by Tsiridis et al. [41] before bone regeneration begins, might explain this.

Bone surface density in osteoporotic rats and connectivity density in all rats showed highest increase after fourteen days. From day fourteen to day twenty-one both parameters decreased. For the ABM/P-15 groups, this development could indicate that new bone initially was formed on the surface of the ABM/P-15 grafting material, then the increase, followed by a new bone formation inside the ABM/P-15 material, and then the decrease. These events could also explain the development in bone surface-to-volume ratio, where an initial increase was observed, followed by a decrease after twenty-one days in both the ABM/P-15 groups in both normal and osteoporotic rats.

Trabecular thickness in the ABM/P-15 groups, both normal and osteoporotic rats decreased significantly from day zero to day fourteen. As a consequence of increased bone tissues, structure types were changed toward a typical plate-like structure, while the structure types in the empty groups remained a typical rod-like structure.

Histologically, substantial bone growth was observed around ABM, indicating that it accelerated bone formation. This might be explained by increased and strengthened cell adhesion due to actin filaments and stress fibres. Furthermore, cells adhered to ABM/P-15 have been shown to increase osteoblastic gene expression for BMP-2, BMP-7, and alkaline phosphatase and to increase matrix deposition [20]. Palmieri et al. [42] demonstrated an upregulation of 11 miRNA genes, involved in osteogenesis and bone remodelling, in human osteoblast-like cells cultured with P-15 peptide. Histological findings in this study indicated that ABM/P-15 had osteoinductive properties [42]. In our study, ABM/P-15 defects had an initial bone volume fraction of 30.2% at day 0, because of the inserted graft material. It was expected that ABM/P-15 would be resorbed over time. By day twenty-one, 9.5% of total defect area volume was ABM/P-15 material in normal rats and 14.6% in osteoporotic rats. Sherman et al. [10] showed 9 ± 7.3% residual of ABM/P-15 in the observation site six months after surgery in an ovine lumbar interbody fusion model. This suggested that the reabsorbing process of ABM/P-15 started within the first weeks of bone healing. Assuming that a part of the graft material has been reabsorbed and replaced by newly formed bone, there could be a tendency to improved bone regeneration in ABM/P-15 treated defect in normal bone.

Taken together, ABM/P-15 accelerated bone regeneration in osteoporotic bone to the same extent as in normal bone on several parameters such as bone volume fraction, surface density, connectivity density, and trabecular thickness suggesting that ABM/P-15 did accelerate bone healing in osteoporotic bone, but not in normal bone.

Despite the above-mentioned literatures, the efficacy of ABM/P-15 on defect repair in vivo longitudinally has not been investigated, more importantly not in an osteoporotic model. Thus, the present study tried to elucidate the effects of ABM/P-15 on bone defect healing in both normal and osteoporotic rat tibias. This design was highly clinically relevant and particularly important for osteoporotic patients. Thus, this study was able to answer to efficacy of ABM/P-15 on bone defect repair and whether results derived from normal bone could be similar to those from osteoporotic bone.

There are several limitations of the study. A group treated with ABM alone (without P-15 coating) could have been used to serve as a control group, which could have helped clarifying the specific effect of the P-15 coating. Additionally, a group treated with autograft would have provided insight to whether ABM/P-15 could be an alternative to autograft as gold standard grafting material for orthopaedic surgery.

Day zero bone volume fraction values in the two empty groups were not identical. This could be a bias in comparing the empty groups. Although the same techniques were used, the bone volume fractions in the two empty groups on day zero were not identical. This small (1.5% versus 2.6%) but significant difference was due to uncleaned bone fragments left inside the holes and could cause a bias in comparing the empty groups.

ABM/P-15 and newly formed bone could not be differentiated with micro-CT scanning, because of overlapping of image grey values with similar density profiles. This could have been a weakness in evaluating new bone formation by micro-CT. We solved this problem by using histology and histomorphometry to evaluate new bone formation at sacrifice. Due to remnants of ABM/P-15 in the defect site, it was not possible to blind the histological analysis of the bone specimens.

The empty groups showed a relative good healing potential in this rat model. Therefore the tibial defects in this study were not critically sized.

In conclusion, this study showed that ABM/P-15 significantly enhanced bone defect repair in a 2.8 mm cylindrical proximal tibia defect in osteoporotic rats, while ABM/P-15 did not accelerate bone defect repair in normal rats.

## Figures and Tables

**Table 1:** 3D microarchitectural properties (mean ± SD) of cortical bone in osteoporosis induction period.

	Cortical porosity (%)	Bone surface density (mm^−1^)	Bone surface-to-volume ratio (mm^−1^)	Pore size (*μ*m)	Cortical thickness (*μ*m)
D −90 (*N* = 12)	2.4 ± 1	5.48 ± 0.53	5.62 ± 0.54	0.03 ± 0.004	0.28 ± 0.02
D −7 (*N* = 12)	2.9 ± 1	4.15 ± 0.51	4.37 ± 0.52	0.04 ± 0.008	0.32 ± 0.02
D0 (*N* = 12)	2.8 ± 1	5.41 ± 0.7	5.69 ± 0.72	0.05 ± 0.02	0.28 ± 0.03
ANOVA	*P* = 0.1794	*P* < 0.001	*P* < 0.001	*P* = 0.002	*P* < 0.001
Difference between groups	NS	D −7 < D −90, D0	D −7 < D −90, D0	D −90 < D −7 < D0	D −7 > D −90, D0

D0 = day 0, D −7 = day −7, and D −90 = day −90 before bone surgery. Values are expressed as mean for group, measured on both hind legs ± standard deviation. NS = no significance. *P* values < 0.05 are considered significant.

**Table 2:** 3D microarchitectural properties (mean ± SD) of cancellous bone in osteoporosis induction period.

	Bone volume fraction (%)	Bone surface density (mm^−1^)	Bone surface-to-volume ratio (mm^−1^)	Structure model index (—)	Connectivity density (mm^−3^)	Trabecular separation (*µ*m)	Degree of anisotropy (—)	Trabecular thickness (*μ*m)
D −90 (*N* = 12)	59.8 ± 5.8	18.84 ± 0.55	31.31 ± 3.88	−2.04 ± 1.37	1234.58 ± 113.57	0.07 ± 0.06	1.37 ± 0.03	0.07 ± 0.004
D −7 (*N* = 12)	20.5 ± 14.1	8.25 ± 5.11	43.78 ± 4.07	3.02 ± 1.01	278 ± 261.42	0.16 ± 0.06	1.45 ± 0.08	0.06 ± 0.003
D0 (*N* = 12)	20.2 ± 15	8.08 ± 5.33	43.97 ± 5.04	3.1 ± 1.18	274.69 ± 262.63	0.16 ± 0.06	1.44 ± 0.09	0.06 ± 0.004
ANOVA	*P* < 0.0001	*P* < 0.0001	*P* = 0.0001	*P* < 0.0001	*P* < 0.0001	*P* = 0.0017	*P* = 0.0265	*P* = 0.0006
Difference between groups	D −90 > D −7, D0	D −90 > D −7, D0	D −90 < D −7, D0	D −90 < D −7, D0	D −90 > D −7, D0	D −90 < D −7, D0	D −90 < D −7	D −90 > D −7, D0

D0 = day 0, D −7 = day −7, and D −90 = day −90 before bone surgery. Values are expressed as mean for group, measured on both hind legs ± standard deviation. NS = no significance. *P* values < 0.05 are considered significant.

**Table 3:** 3D microarchitectural properties (mean ± SD) in proximal tibial defects in normal rats.

	Bone volume fraction (%)	Bone surface density (mm^−1^)	Bone surface-to-volume ratio (mm^−1^)	Structure model index (—)	Connectivity density (mm^−3^)	Trabecular separation (*μ*m)	Degree of anisotropy (—)	Trabecular thickness (*μ*m)
Empty								
D0 (*N* = 9)	1.5 ± 1.9	0.61 ± 0.56	83.8 ± 26.3	5.7 ± 0.99	16 ± 29.5	0.96 ± 0.56	1.19 ± 0.1	0.04 ± 0.03
D7 (*N* = 9)	6.3 ± 3.2	4.32 ± 1.71	91.3 ± 18.1	4.43 ± 0.56	146.3 ± 116.8	0.25 ± 0.06	1.08 ± 0.03	0.04 ± 0.02
D14 (*N* = 9)	27.8 ± 8.5	15.88 ± 3.07	62.4 ± 10.1	2.41 ± 0.72	1246.3 ± 321.4	0.09 ± 0.03	1.13 ± 0.03	0.05 ± 0.01
D21 (*N* = 9)	38.2 ± 11.8	14.41 ± 2.83	40.2 ± 9.5	1.66 ± 1.32	851.4 ± 239.4	0.09 ± 0.02	1.11 ± 0.04	0.07 ± 0.01
ANOVA	*P* < 0.001	*P* < 0.001	*P* < 0.001	*P* < 0.001	*P* < 0.001	*P* < 0.001	*P* = 0.002	*P* = 0.034
Difference between groups	D0 < D7 < D14, D21	D0 < D7, D14, D21	D14, D21 < D0, D7	D0 > D7 > D14, D21	D0, D7 < D21 < D14	D0 > D7 > D14, D21	D0 > D7	NS

ABM/P-15								
D0 (*N* = 9)	30.2 ± 9.7	8.17 ± 2.26	28.3 ± 2.3	6.14 ± 1.59	333.2 ± 193.4	0.12 ± 0.03	1.11 ± 0.03	0.13 ± 0.01
D7 (*N* = 9)	25.6 ± 3.5	9.36 ± 1.29	38.4 ± 2.2	5.58 ± 0.93	529.4 ± 175.3	0.13 ± 0.02	1.08 ± 0.03	0.12 ± 0.01
D14 (*N* = 8)	44.8 ± 4.2	17.39 ± 1.17	39.6 ± 1.8	2.19 ± 0.7	1506.8 ± 219.3	0.07 ± 0.01	1.12 ± 0.03	0.1 ± 0.004
D21 (*N* = 9)	56 ± 5.7	15.88 ± 0.91	28.6 ± 2.9	0.4 ± 1.49	1173 ± 155.9	0.06 ± 0.01	1.1 ± 0.02	0.11 ± 0.01
ANOVA	*P* < 0.001	*P* < 0.001	*P* < 0.001	*P* < 0.001	*P* < 0.001	*P* < 0.001	*P* = 0.037	*P* < 0.001
Difference between groups	D0, D7 < D14 < D21	D0, D7 < D14, D21	D0, D21 < D7, D14	D0, D7 > D14 > D21	D0, D7 < D21 < D14	D0, D7 > D14, D21	D7 < D14	D0 > D7, D21 > D14

D0 = day 0, D7 = day 7, D14 = day 14, and D21 = day 21 after surgery. NS = no significance. *P* values < 0.05 are considered significant.

**Table 4:** 3D microarchitectural properties (mean ± SD) in proximal tibial defects osteoporotic rats.

	Bone volume fraction (%)	Bone surface density (mm^−1^)	Bone surface-to-volume ratio (mm^−1^)	Structure model index (—)	Connectivity density (mm^−3^)	Trabecular separation (*μ*m)	Degree of anisotropy (—)	Trabecular thickness (*μ*m)
Empty								
D0 (*N* = 12)	2.6 ± 1.4	1.15 ± 0.57	59.2 ± 13.2	6.5 ± 1.26	22.7 ± 18.8	0.39 ± 0.22	1.14 ± 0.05	0.06 ± 0.02
D7 (*N* = 12)	8.8 ± 3.5	5.38 ± 2.2	81.6 ± 16.4	4.73 ± 0.65	229.7 ± 190.8	0.2 ± 0.03	1.09 ± 0.04	0.05 ± 0.01
D14 (*N* = 12)	22.7 ± 6.4	10.95 ± 2.44	51.6 ± 7.2	2.57 ± 0.64	627 ± 202.5	0.13 ± 0.02	1.09 ± 0.02	0.05 ± 0.01
D21 (*N* = 12)	20 ± 8.7	7.43 ± 3.56	38.3 ± 6.3	2.81 ± 0.71	313.5 ± 263.7	0.18 ± 0.05	1.12 ± 0.04	0.07 ± 0.01
ANOVA	*P* < 0.001	*P* < 0.001	*P* < 0.001	*P* < 0.001	*P* < 0.001	*P* < 0.001	*P* = 0.001	*P* = 0.001
Difference between groups	D0 < D7 < D14, D21	D0 < D7, D21 < D14	D21 < D0, D14 < D7	D0 > D7 > D14, D21	D0 < D7, D21 < D14	D14 < D0, D7, D21	D0 > D7, D14	D21 > D7, D14

ABM/P-15								
D0 (*N* = 12)	31.5 ± 6.1	8.77 ± 1.38	28.9 ± 2.2	6.33 ± 1.2	365.6 ± 128	0.11 ± 0.01	1.1 ± 0.03	0.13 ± 0.004
D7 (*N* = 12)	31.2 ± 7.2	10.23 ± 1.93	34.9 ± 4.9	5.91 ± 1.21	564 ± 232.9	0.11 ± 0.02	1.08 ± 0.04	0.12 ± 0,01
D14 (*N* = 12)	51.8 ± 5.8	16.38 ± 1.27	32 ± 4.1	1.4 ± 1.04	1348.2 ± 196.2	0.07 ± 0.01	1.08 ± 0.03	0.11 ± 0,01
D21 (*N* = 12)	57 ± 5.9	14.31 ± 1.23	25.3 ± 3.3	0.7 ± 1.25	969.1 ± 146.5	0.07 ± 0.01	1.07 ± 0.03	0.12 ± 0,01
ANOVA	*P* < 0.001	*P* < 0.001	*P* < 0.001	*P* < 0.001	*P* < 0.001	*P* < 0.001	*P* = 0.296	*P* < 0.001
Difference between groups	D0, D7 < D14, D21	D0 < D7 < D21 < D14	D21 < D7, D14	D0, D7 > D14, D21	D0, D7 < D21 < D14	D0, D7 > D14, D21	NS	D0 > D7, D14

D0 = day 0, D7 = day 7, D14 = day 14, and D21 = day 21 after surgery. NS = no significance. *P* values < 0.05 are considered significant.
